# Assigning NMR spectra of RNA, peptides and small organic molecules using molecular network visualization software

**DOI:** 10.1007/s10858-019-00271-3

**Published:** 2019-07-19

**Authors:** Jan Marchant, Michael F. Summers, Bruce A. Johnson

**Affiliations:** 1 Department of Chemistry and Biochemistry, University of Maryland, Baltimore County, 1000 Hilltop Circle, Baltimore, MD 21250 USA; 2 Howard Hughes Medical Institute, University of Maryland, Baltimore County, 1000 Hilltop Circle, Baltimore, MD 21250 USA; 3 Structural Biology Initiative, CUNY Advanced Science Research Center, 85 St. Nicholas Terrace, New York, NY 10031 USA

**Keywords:** RNA, Peptides, Chemical shift assignment, Prediction, Software

## Abstract

NMR assignment typically involves analysis of peaks across multiple NMR spectra. Chemical shifts of peaks are measured before being assigned to atoms using a variety of methods. These approaches quickly become complicated by overlap, ambiguity, and the complexity of correlating assignments among multiple spectra. Here we propose an alternative approach in which a network of linked peak-boxes is generated at the predicted positions of peaks across all spectra. These peak-boxes correlate known relationships and can be matched to the observed spectra. The method is illustrated with RNA, but a variety of molecular types should be readily tractable with this approach.

**Electronic supplementary material:**

The online version of this article (10.1007/s10858-019-00271-3) contains supplementary material, which is available to authorized users.

The power of NMR spectroscopy relative to other molecular spectroscopies lies in the ability to detect spectral signals and interactions associated with specific atoms. Requisite assignment of NMR signals typically follows a paradigm of measuring the chemical shifts of local maxima (peaks) within each spectrum followed by correlating signals within and among different types of NMR spectra and associating those peak positions with specific atoms, either by automated methods or interactive analysis. Although automated assignment methods are desirable and work toward this goal is ongoing, interactive methods remain the standard for much NMR assignment. Although interactive analysis is aided by the display of peak-boxes associated with each measured peak, which can display known assignments or other annotations, the tracking of thousands of scalar- and dipolar-coupled peaks in multiple datasets can be challenging. We describe here an inverted approach that focuses on networks of coupled peaks that are predicted from the molecular structure and type of NMR experiment. Instead of picking peak positions and then attempting to assign them, we generate a linked network of assigned peak-boxes at these predicted positions that can then be interactively aligned with the observed spectra. This approach allows the spectroscopist to make simultaneous use of multiple spectral features that can minimize ambiguity in the assignments compared to the process of assigning individual peaks.

Our technique relies on a priori knowledge of the molecular topology and the ability to predict chemical shifts and coupling patterns. Both types of information are available for a range of molecule types (Steinbeck et al. 2003; Ulrich et al. 2008; Barton et al. 2013; Brown et al. 2015). We have used the technique to assign RNAs as large as 68 nucleotides (including fragments of much larger RNA projects) (Keane et al. 2015; Marchant et al. 2018; Zhang et al. 2018) and small cyclic peptides, but the principles apply to many molecular types including DNA, modest sized peptides, and arbitrary small organic molecules. The higher the quality of chemical shift predictions and predicted NOE peaks the better the starting point, but the approach allows bootstrapping from better portions of the starting set to regions of lower quality.

The approach described here could be implemented with a variety of tools for chemical shift prediction, peak network generation and interactive assignment. Here we describe using the protocol with NMRFx Analyst, a software tool that is freely available and open sourced and extends the existing NMRFx Processor (Norris et al. 2016). NMRFx Analyst integrates NMR processing, chemical shift prediction and peak picking and assignment tools useful for this approach. An earlier implementation of the approach is also available in NMRViewJ (Johnson and Blevins 1994). There are several requirements to implement the approach in other tools. The key requirements are a source of predicted shifts and the ability to interactively move multiple peaks in response to the movement of a single peak. The shift prediction is done once at the start of a project, and so can be done with an external tool. Generation of peaks based on the NMR shift prediction can also be done external to the software. So any NMR analysis tool (such as CCPN Analyst (Skinner et al. 2016), Sparky (Lee et al. 2015) or CARA(Keller)) that can read external peak files has the core technology to get started without any modifications. The interactive adjustment of peak positions in response to moving a single peak would likely require code modifications or a plugin module, but this should be relatively straightforward to implement.

We describe here the approach for a 50-nucleotide RNA hairpin. A 3 min video illustrating the major steps on a 22-nucleotide RNA hairpin is available as supplementary material. The molecular topology for the RNA is readily available from the primary sequence coupled with NMRFx Analyst’s built-in library of nucleotides. A secondary structure (or if available, the tertiary structure) is additionally necessary for chemical shift prediction and NOE cross-peak prediction. Predicting chemical shifts of the target molecule is an essential component of the protocol. For RNA molecules we use our previously described attribute-based shift prediction technique (Barton et al. 2013; Brown et al. 2015), but with 3D coordinates a structure based method could be used (Frank et al. 2013, 2014; Brown et al. 2015). The attribute technique predicts hydrogen, carbon, and nitrogen chemical shifts based on a set of attributes describing the central nucleotide in a five-nucleotide window. The only input necessary is the primary sequence and a dot-bracket style representation of the secondary structure (Lorenz et al. 2011).

For RNA assignments we have used a set of three different experiments. These are homonuclear 2D TOCSY, 2D ^1^H-^13^C HMQC and 2D NOESY. The technique is not dependent on having the TOCSY and HMQC experiments, but a greater number of complementary experiments will reduce ambiguities in the assignment process. Each experiment type necessitates a different protocol for peak-box generation. The TOCSY protocol simply generates peak-boxes for protons that have less than a specified number of homonuclear J-coupling steps. In particular, the H5–H6 coupling of uracil and cytosine and couplings between ribose protons are generated. The HMQC involves all carbons with directly bonded protons. While the expected peaks and correlations for the HMQC and TOCSY are relatively insensitive to tertiary structure, peak-box generation for the NOESY involves various assumptions.

For an RNA (or other molecule) where the 3D structure is known peak-boxes are generated for all hydrogen pairs whose distance is less than a specified limit (often 5 or 6 Å). Where the 3D structure is not available, NMRFx Analyst uses the secondary structure specified with dot-bracket notation and a built-in set of rules to generate peak-boxes for helical and tetraloop regions. Intra-residue peak-boxes are also generated and are less dependent on the structural information. While this NOESY protocol is unable to generate inter-residue peak-boxes in larger loops, the combination of peak-boxes in helical and tetraloop regions and intra-residue peak-boxes in all regions gives a substantial number of predicted peaks that can be used as a basis for a search to other regions. The intra-residue assignments can be used to get the correct shift assignments which are then used to assign peaks that haven’t been predicted (*vide infra*).

Overlapping peaks are a serious impediment to the assignment of larger RNAs, but this can be alleviated by the use of isotopically labeled RNA molecules to minimize the number of spectral peaks (Lu et al. 2010; Longhini et al. 2016). Nucleotide and atom-specific ^2^H labeling, or ^13^C labeling combined with pulse sequences that filter and edit the spectra based on the presence of ^13^C labelled nuclei can be used to generate a complementary set of experiments in which the number of peaks in each individual experiment is reduced, but all expected peaks can be observed in the complete set of experiments (LeBlanc et al. 2017). NMRFx Analyst allows specifying the labeling pattern by both nucleotide type and specific residues. The peak-box generator uses this in combination with each experiment’s edit-filter scheme to generate the expected cross-peaks for the labelled RNA.

Once the set of peak-boxes is generated for each experiment the user can begin to interactively assign the spectra. Each available spectrum is displayed with its corresponding peak-boxes superimposed. Any given spectrum might be displayed in multiple windows so that expansions of relevant portions of the spectra can be displayed. The user can then interactively drag, with motions of mouse or track pad, a peak-box from its predicted position to alignment with an observed spectral peak (Fig. 1).

**Fig. 1:**
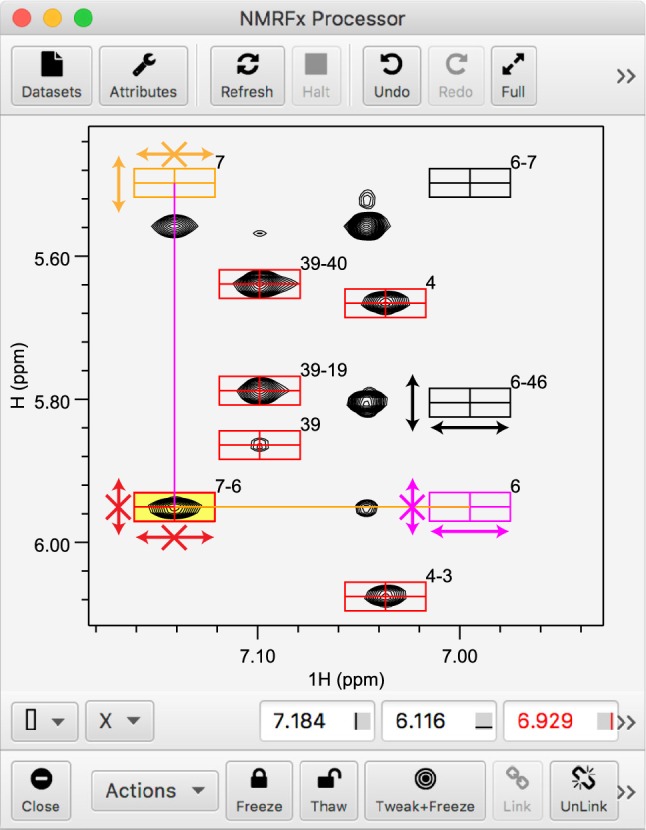
Screenshot of the NMRFx Analyst GUI with a network assignment procedure in progress. The rectangular peak-boxes illustrate predicted peaks, label numbers indicate the residues involved, and arrows are used to show whether peak-boxes can be moved in each dimension (no X) or are frozen in that dimension (with X). Peak-boxes in black (with residue numbers 6–46, and 6–7) are initially in the predicted positions and can be freely adjusted, as shown by black arrows for peak-box 6–46. Peak-box 7–6 (red) has been selected (yellow background) and then frozen and can no longer be adjusted in either dimension. As a consequence of freezing this peak-box, peak-box 7 (orange) is now frozen in the horizontal position yet adjustable in the vertical so it could be slid down to align with the peak below. The opposite is true for peak-box 6 (magenta) which could be slid left to align with a peak. Other red peak-boxes have already been positioned and frozen. Controls at bottom allow for freezing and thawing peaks. The Tweak + Freeze button will automatically center a peak-box on an overlapped peak before freezing

In the traditional approach, peak-boxes are initially not assigned so there is no unambiguous relationship between different peak-boxes within the spectrum or between spectra. In this new approach, while peak-boxes are not necessarily correctly positioned, they each have an assigned atom for each dimension. The assignment means that sets of peaks will share atoms on one or both dimensions. This is illustrated visually when one selects a peak as shown in Fig. 1. Connecting lines are drawn between peak-boxes with common atom assignments. As a user drags a peak-box, the entire set of peak-boxes that share an atom with the moved peak will move synchronously with the directly shifted peak. The essence of the method is that whereas observing an individual peak in relation to a spectral signal might be ambiguous, a whole set of coupled peaks is not.

Individual peak-boxes may initially be predicted to be close to multiple spectral signals, precluding unambiguous placement in isolation. In this new approach, however, the entire set of linked peak-boxes across multiple experiments inform the user’s decision. An example of this is shown in Fig. 2, step 3, where two possible alignments of a group of peak-boxes within the NOESY spectrum are possible, but can be resolved with analysis of the HMQC spectrum. Positioning peak-boxes in crowded regions is still difficult, but is often unnecessary due to the presence of linked peak-boxes that are in uncrowded regions. An additional practical advantage of the approach is that typographical errors are minimized. Rather than the user typing in, with possible errors, an atomic assignment to a peak-box label field in the GUI, all peaks start with a computer generated assignment.

**Fig. 2:**
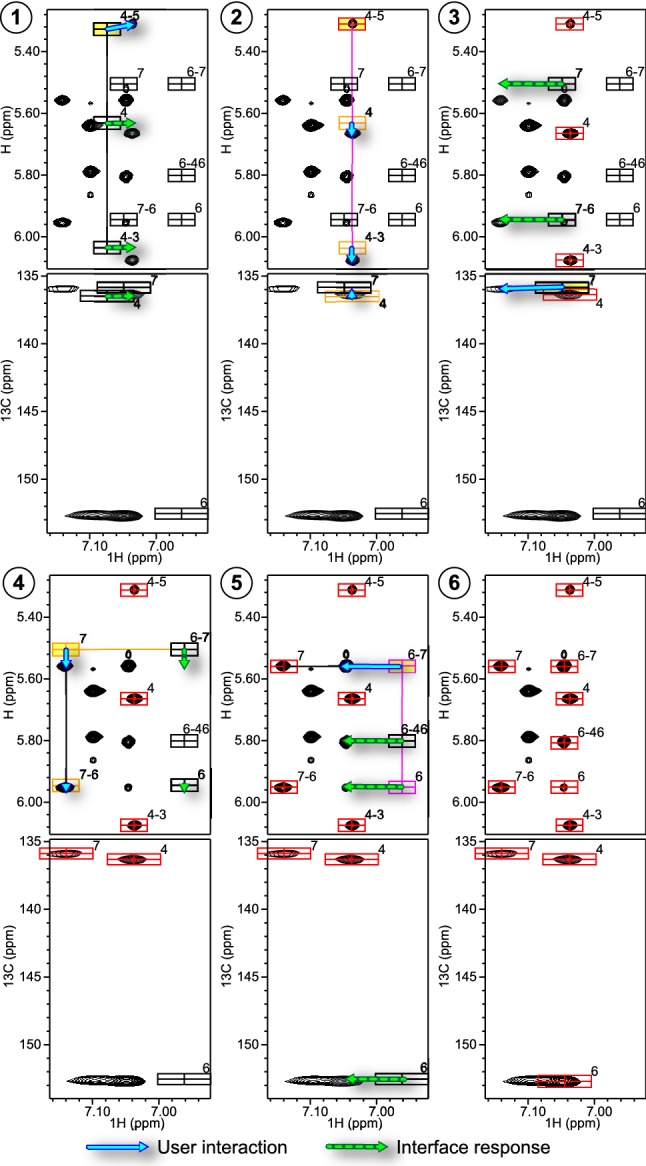
Demonstration of the assignment procedure for a portion of a 50 nt RNA. In each panel the upper spectrum is a ^1^H-^1^H NOESY and the lower a ^1^H-^13^C HMQC. **1** Peak-boxes are initially positioned according to predicted chemical shifts. Upon selecting a peak-box for positioning, the linked peak-boxes are indicated by connecting lines. Visual inspection identifies a candidate peak to which the peak-box labeled 4–5 is manually repositioned, as indicated by the solid arrow. Linked peaks are repositioned automatically, as indicated by the dashed arrows. **2** The peak-box position is frozen, indicated in red. The remaining three peak-boxes in the spin system are automatically frozen, and prevented from moving in their shared dimension, indicated in orange for the *x*-axis. Their associated peaks are readily identified due to this restriction. **3** Examination of the NOESY spectrum reveals two well-matched possibilities for assignment of the peak-box labeled 7. The correct assignment is found by reference to the HMQC spectrum, in which there is only one reasonable candidate. **4** Repositioning the remaining peak-boxes for the spin-system associated with this atom automatically repositions associated peak-boxes from the remaining spin-system under consideration. **5** The remaining spin-system contains peak-boxes restricted from moving along the *y*-axis due to previously frozen peaks, indicated in magenta, such that their associated peaks are readily identified. **6** Final positions of the peak-boxes under consideration

The protocol is greatly facilitated by a means to specify whether any given peak-box has been positioned into a final location. In NMRFx Analyst, this is done by clicking a “Freeze” button or using a corresponding keyboard shortcut. Once frozen, a peak-box will be displayed with a different color so that the user has a visual indication of which peak-boxes have been confidently placed (Fig. 1). Freezing an individual peak-box will lock both of its dimensions to their current position so that it can’t subsequently be moved. The linked (sharing the same atom) dimensions of other peak-boxes, in the same and different experiments, will also be frozen. Thus, linked peak-boxes might only be frozen in a single dimension. Such peak-boxes may only be slid along the free dimension which facilitates their assignment by minimizing the choice of locations to a single dimension. A color scheme is used to indicate whether a peak is frozen on the x-axis, y-axis or both axes. Peak-boxes can also be unlocked via a “Thaw” button. Freezing peak-boxes also updates the atom assignment table with the chemical shift of the peak-box dimensions. Thus the final assignment list is generated from only peak-boxes that have been frozen.

As described above, the set of peak-boxes generated for NOESY spectra requires assumptions about the molecular structure and it is unlikely that they will perfectly match the spectra. Extraneous peak-boxes are easily deleted. Where peaks cannot be associated with a generated peak-box, the user can manually add a peak-box at the peak’s location. The software still provides significant value in this process as the observed signal might align with peak-boxes that have already been frozen. In this case assignment possibilities for the manually added peak-box are displayed and a link can be made to the already frozen peak-boxes.

The above description has focused on applications to RNA. The approach, however, was initially developed as a means to assign cyclic peptides. The basic protocol for peptides is essentially the same as described above. The differences involve methods for chemical shift prediction and rules for peak-box generation. Predicted chemical shifts can be obtained simply from average chemical shifts for standard amino-acids available from the BMRB (Ulrich et al. 2008). Alternatively, NMRFx Analyst includes a built-in (as yet, unpublished) tool for generating predictions based on sequence and dihedral angles, and optionally ring-current shifts. Projects involving cyclic peptides often include non-canonical amino-acids (Hosseinzadeh et al. 2017). Shift prediction for non-canonical amino-acids is supported using a built-in predictor based on HOSE codes that can form predictions for any arbitrary organic molecule. Peptides, and all other molecules supported, can also use predictions generated in 3rd party software and imported from a text file. As for RNA, 2D TOCSY, ^1^H-^13^C HMQC and 2D NOESY experiments have been implemented, but various experiment combinations are possible. COSY experiments can be included, for example, by using the TOCSY peak-box generation protocol but limiting the number of transfer steps in the peak generator to one. The TOCSY and HMQC experiments are particularly robust because they don’t depend on having 3D structural information, though constraints involved in cyclizing the peptide can be used to generate a reasonable family of structures for NOESY predictions.

The described protocol is also completely applicable to arbitrary small organic molecules and provides a means to rapidly assign, without typographical errors, these molecules using one or more 2D spectra. Predictions can be made using the internal HOSE code based predictor or external tools (Schütz et al. 1997; Smurnyy et al. 2008). Prediction of NOESY peaks to complement those from scalar-coupled experiments can be made with an approximate 3D structure. Missing and additional peaks can be dealt with as described above.

While the chemical shift predictions that are used always have some level of error, a key benefit of this approach is that individual errors of large magnitude are easily identified and tolerated due to redundancy in the network of moving peaks. More widespread errors in the predicted chemical shifts, particularly if accompanied by errors in the predicted network of NOEs, would potentially prove more challenging, however in our experience of close to 100 distinct RNA molecules this problem has not arisen. This tolerance to error should also allow the method to be used in situations such as RNA–protein complexes where the RNA chemical shifts near the interface are perturbed from their expected values.

The above protocol, as implemented in NMRFx Analyst, provides a rapid way to facilitate the assignment of a variety of RNA, DNA, peptides and small molecules. It has been used for the assignment of a variety of published RNA projects (Keane et al. 2015; Marchant et al. 2018; Zhang et al. 2018) and for rapid assignment of a variety of cyclic peptides (unpublished studies). Its use requires access to chemical shift predictions which are available within NMRFx Analyst or through a wide variety of external software packages. Prediction of peaks expected in scalar-coupled experiments (e.g. TOCSY, COSY, and HMQC) require only an understanding of the covalent structure of the molecule and prediction of a significant number of NOESY peaks can be made with reasonable assumptions about structure. In particular, intra-residue peaks can be predicted and used to aid in assigning inter-residue peaks. The protocol fits between the traditional manual assignment methods that rely on assigning picked peaks and fully automated methods. We anticipate that it will form a basis for adding more automated capabilities in the future. For example, one can already drag a peak near to a signal and have it automatically positioned to the close peak. By basing the automated capabilities on this visual tool, the user will be able to observe the results of the automation and manually intervene. As chemical shift and structural prediction methods are developed across all molecule types, we expect the approaches for chemical shift assignment illustrated here to be adopted into widespread use.

## Electronic supplementary material

Below is the link to the electronic supplementary material.

Supplementary material 1 (MP4 22476 kb)
